# Oxidative stress promotes autophagic cell death in human neuroblastoma cells with ectopic transfer of mitochondrial PPP2R2B (Bβ2)

**DOI:** 10.1186/1471-2121-10-91

**Published:** 2009-12-18

**Authors:** Wan-Ting Cheng, Zhi-Xuan Guo, Chia-An Lin, Ming-Yi Lin, Li-Chu Tung, Kang Fang

**Affiliations:** 1 Department of Life Science, National Taiwan Normal University, Taipei, Taiwan

## Abstract

**Background:**

The multifunctional protein phosphatase 2A (PP2A) is a heterotrimeric serine/threonine protein phosphatase composed of a scaffolding, catalytic and regulatory subunits. By modifying various downstream signal transducers, the aberrant expression of the brain-targeted regulatory subunit PPP2R2B is associated with the onset of a panel of neuronal disorders. The alternatively splicing of PPP2R2B encodes two regulatory subunit isoforms that determine cellular distribution of the neuron-specific holoenzyme to mitochondria (Bβ2) and cytoplasm (Bβ1), respectively.

**Results:**

Human neuroblastoma cells were transfected with *PPP2R2B *constructs encoding the complete sequences of Bβ2 and Bβ1, respectively. The colonies with antibiotic resistance were selected as stable cell lines. Both ectopic Bβ1 and Bβ2 clones exhibited characteristics of autophagy. To test how cells respond to reactive oxygen species generators, the cells were treated with either hydrogen peroxide or t-butyl hydroperoxide and Bβ2 clones induced cell death. Suppression of autophagy using either RNA interference of the essential autophagy gene or pharmacological inhibitor rescued cell death caused by oxidative stress.

**Conclusions:**

Cells with ectopically expressed mitochondria-targeted regulatory subunit PPP2R2B of the holoenzyme PP2A were shown predisposed to autophagy and oxidative stress induced cell death that is related to apoptosis. The results promised a model for studying the mechanism and function of aberrant PPP2R2B expression in neuronal cells. The work provided a new target for understanding and prevention of neuropathogenesis.

## Background

Protein phosphatase 2A (PP2A) regulates cellular proliferation and tissue growth by antagonizing protein kinases and plays an important role in controlling cell cycle checkpoints, regulating nuclear telomerase activity and modulating dephosphorylation of neurofilaments [1-5]. It is believed that the enzyme flexibility and the substrate specificity are determined by the regulatory subunit (B) that binds to the ubiquitous catalytic (C) and scaffolding (A) subunits [6] to form the heterotrimeric PP2A.

The regulatory B subunits can be categorized into three distinct families based on their homology, namely B (B55 or PR55), B' (B56 or PR61), B"(PR48/59/72/130) and B' " (PR93/110). Furthermore, each B family is composed of several isoforms from different genes [7,8]. There is no sequence or structural similarities among the three gene families. The brain-specific regulatory B subunit, PPP2R2B (Bβ), is widely expressed in the neurons of brain and cerebellum and consists of tryptophan-glutamate repeat-containing β-propeller proteins. The various N termini of B subunit determine enzyme activity, subcellular localizations and neuronal functions of PP2A [3,9,10]. Mainly expressed in Purkinje cells of the cerebellar cortex [5,11,12] and in tissues of spinocerebellar ataxia [13,14], PPP2R2B is also known in mediating PP2A-regulated dephosphorylation of several substrates; they include vimentin [15] and histone-1 [16] as well as microtube-associated protein in neuron cells [17].

The alternative splicing of PPP2R2B generates two major isoforms. They include Bβ1 and Bβ2 with two distinct N terminal tails [5]. The different N termini with 21 and 24 residues of Bβ1 and Bβ2 each encode distinctive subcellular signals that target PPP2R2B isoforms to cytoplasm and mitochondria, respectively, and affect cellular phosphatase activity [18]. The up-regulated mitochondrial Bβ2 was reported promoting apoptosis in response to deprived growth factors [19], while an elevated expression of cytosolic Bβ1 due to CAG repeat expansion at the 5' end of the gene causes autosomal dominant disease of spinocerebellar ataxia type 12 (SCA12) [20].

The aberrant expression of brain-specific PPP2R2B affecting PP2A activity has been implicated in cerebellar atrophy [12]. Translocation of PPP2R2B to mitochondria promotes mitochondria fission, apoptosis [21] and neuronal differentiation [19]. Being implicated in neurodegenerative diseases, oxidative stress causes neuron cell damage and reduction of viable cells. How cells with overexpressed PPP2R2B react to environmental stress remains unclear. Mitochondria are the principal targets for reactive oxygen species (ROS). To address this, the work began with generation of stable cell lines by transferring *Bβ1 *and *Bβ2*, respectively, into human neuroblastoma SK-N-SH cells. The stable clones targeting ectopic PPP2R2B to mitochondria or cytoplasm exhibit characteristics of autophagy that is distinct from the parental cells. Furthermore, only Bβ2 clones were sensitive to ROS treatment and susceptible to autophagy-mediated cell death. The resulting apoptosis can be suppressed by chemical inhibitor and RNA interference of the related autophagy gene, and thereby preventing cells from ROS damage-induced cell death. The work illustrates the significance of autophagy in maintaining viabilities of neuronal cells with ectopically transferred PPP2R2B as well as in accelerating pathogenesis when encountered with oxidative stress.

## Results

### The establishment of clones targeting B*β*2 to mitochondria and B*β*1 cytoplasma, respectively

**Figure 1:**
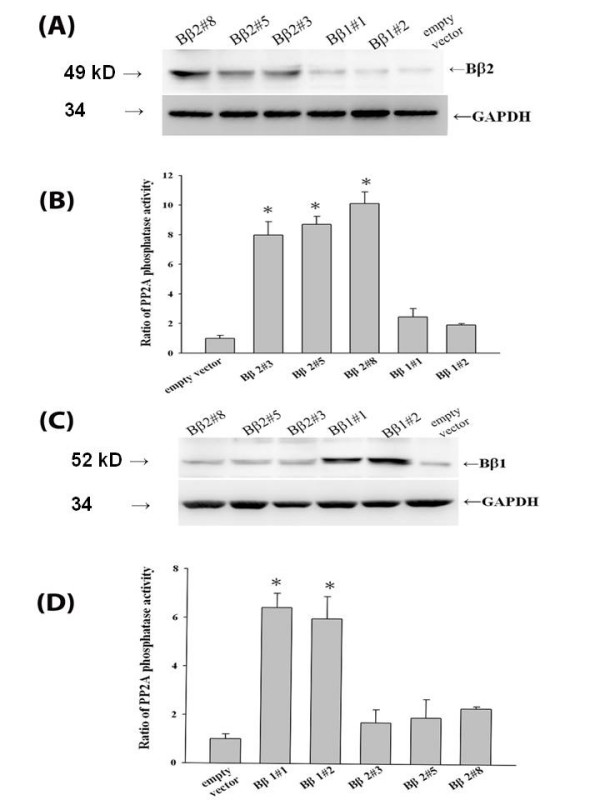
**Both immunoactive Bβ1 and Bβ2 clones acquired enhanced PP2A activity (A)**. Cell lysates of SK-N-SH cells, Bβ1 and Bβ2 clones were probed with signal sequence-specific Bβ2 antiserum. The blots were detected with ECL system following incubation with secondary HRP-conjugated goat against rabbit antibody. The ectopic expressions of Bβ2 were detected Bβ2 (A) clones. Each blot was reprobed with glucose-6-phosphate dehydrogenase (GAPDH) antibody to confirm equal loading of the proteins. (B) The PP2A phosphatase activities of Bβ2 clones were determined by immunoprecipitating cell lysates with Bβ2 antisera as described in Materials and Methods. **Characterizations of clones Bβ1#1 and Bβ1#2 (C) **Cell lysates of SK-N-SH cells, Bβ1 and Bβ2 clones were probed with Bβ1 antiserum and reprobed with GAPDH antibody to confirm equal loading of the proteins. The blots were detected with ECL system. **(D) **The PP2A phosphatase activities of Bβ1 clones were determined by immunoprecipitating cell lysates with Bβ1 antisera as described before. The results represented fold of increase of activity relative to control of SK-N-SH with empty vector shown as average values in three individual experiments. The result represented mean values of three individual determinations; the bars standard errors in three independent experiments as conducted. *P < 0.05, significance of difference as compared with the control group.

The expression of B family regulatory subunits regulates cell developmental patterns in brain [10]. The antibiotic-resistant clones from human neuroblastoma cells, SK-N-SH, through ectopic transfection of either *Bβ1 *or *Bβ2 *cDNA' s driven by cytomegalovirus promoter were established as stable cell lines that can be propagated in serum-supplemented media. In addition to the basal levels in SK-N-SH cells, three isolated clones exhibited increased immune-active signals by sequence-specific Bβ2 antiserum (Bβ2#3, Bβ2#5 and Bβ2#8, Fig 1A), while two were detected by Bβ1 antiserum (Bβ1#1 and Bβ1#2, Fig 1C). A parallel increase of PP2A activities in the immunoprecipitates was also observed in the selected Bβ2 (Fig 1B) and Bβ1 (Fig 1D) clones that corresponded with their increased sensitivities to antisera in western blot. In contrast to the parental cells, the stable Bβ2 clones tend to aggregate to form clusters in culture (data not shown).

**Figure 2:**
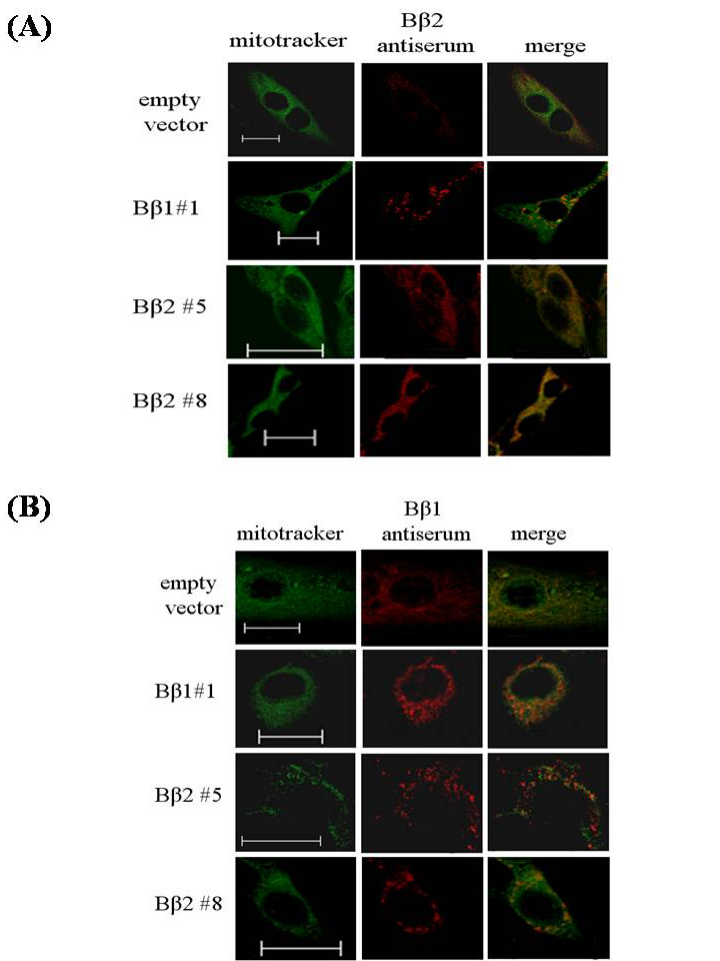
**Differential subcellular localization of ectopic Bβ2 and Bβ1 by confocal image analysis**. **(A) **Cells were immunostained with antiserum against β2 (*red*) and counter-stained with mitotracker (*green*) and visualized by confocal microscopy. The merged graph showed that β2 was colocalized as yellow colors at mitochondria in clone Bβ2 #5 and #8. (B) Cells were immunostained with antiserum against β1 (*red*) and mitotracker as before. The merged graph showed that ectopic β1 can be distributed in cytoplasm in clone Bβ1#1 as compared with SK-N-SH cells. *Bar*, 20 μm.

The strong signals in clones by Bβ2 antiserum overlapping with mitotracker green marked direct targeting of ectopic Bβ2 at mitochondria (Fig 2A); while little overlay was detected between mitochondria and the increased signal as determined by Bβ1 antiserum indicated cytoplasm distribution of Bβ1 in the isolated clones (Fig 2B). Despite some degree of non-specific staining by the antisera, the results provided evidence that stable clones by targeting cytoplasm with ectopic Bβ1 and mitochondria with Bβ2, respectively, can be achieved.

### The enhanced autophagosome formation in clones with ectopic PPP2R2B

**Figure 3:**
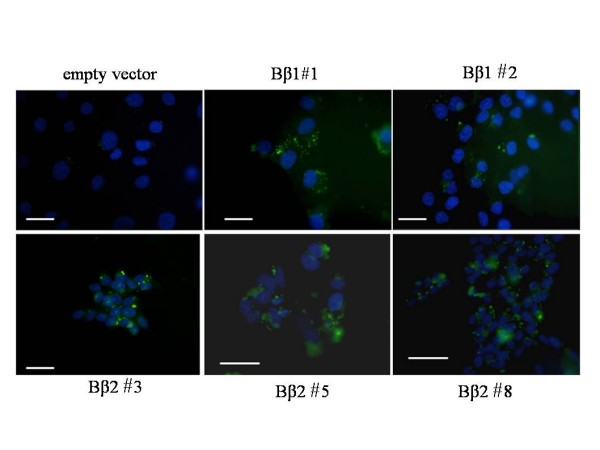
**Autophagosome formation expressing GFP-LC3 puncta in Bβ2 and Bβ1 clones**. The parental SK-N-SH cells and Bβ1 and Bβ2 clones were transfected with GFP-LC3 plasmid for 48 h and observed under the fluorescence microscope for GFP (*green*) followed by counter-staining with DAPI(*blue*). Puncta fluorescence in GFP-LC3 transfected cells can be detected in cells of both Bβ1 and Bβ2 clones. *Bars*, 20 μm.

An increased autophagy has been reported in human cells or tissues with neuronal injury [22,23]. We then assessed whether coordinated regulation of mitochondrial networks and autophagy-regulated cell viability can be recapitulated in clones with acquisition of PPP2R2B functions. As LC3 is modified with the lipid phospatidylethanolamine before being located to autophagosomes [24], the emitted fluorescence can be used to monitor autophagosome formation in cells through transient transfection with expression vector, green fluorescent protein-tagged light chain 3 (GFP-LC3). The results showed that GFP puncta that represented autophagy development can be detected in both Bβ1 and Bβ2 clones, but not in the parental cells (Fig 3). Thus, the presence of autophagosome is closely related to ectopically expressed PPP2R2B.

### Sensitivity of peroxide-induced apoptotic cell death is associated with ectopic B*β*2

**Figure 4:**
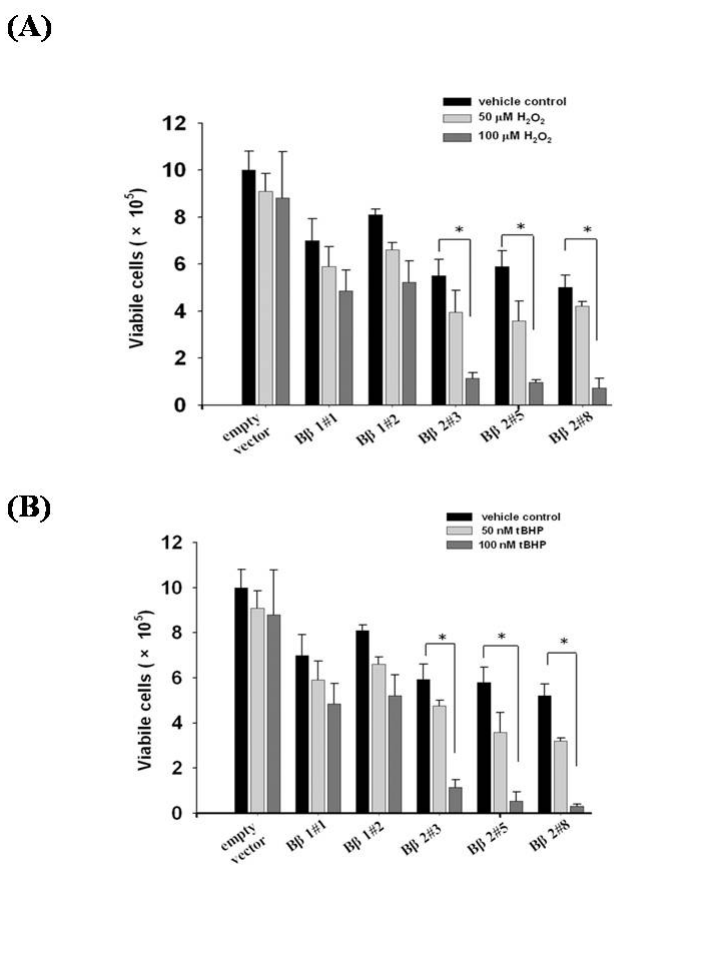
**Cells growth curves as affected by various concentrations of H_2_O_2 _and tBHP**. The exponentially growing SK-N-SH cells or Bβ1 and Bβ2 clones were seeded at a total of 2 × 10^5 ^cells per 6-cm plate and treated with different concentrations of H_2_O_2 _(0, 50 and 100 μM, respectively) (A) or tBHP (0, 50 and 100 nM, respectively) (B). After 48 h of treatment, the cells were trypsinized and counted by trypan blue exclusion assay. The result represented mean values of three individual determinations; the bars standard errors in three independent experiments as conducted. *P < 0.05, significance of difference as compared with the control group.

The two ROS generators H_2_O_2 _(Fig 4A) and *t*-butyl hydroperoxide (tBHP) (Fig 4B) suppressed growth in Bβ2 clones in dose-dependent manners, while both Bβ1 clones and SK-N-SH cells with empty vector alone were not affected. Compared with H_2_O_2_, the suppression of viable cells was more evident in tBHP that exerted more sensitivity in cells with ectopic Bβ2. The escalated cell death is proportional to the levels of Bβ2, in which Bβ2#8 was more sensitive to peroxide treatment.

**Figure 5:**
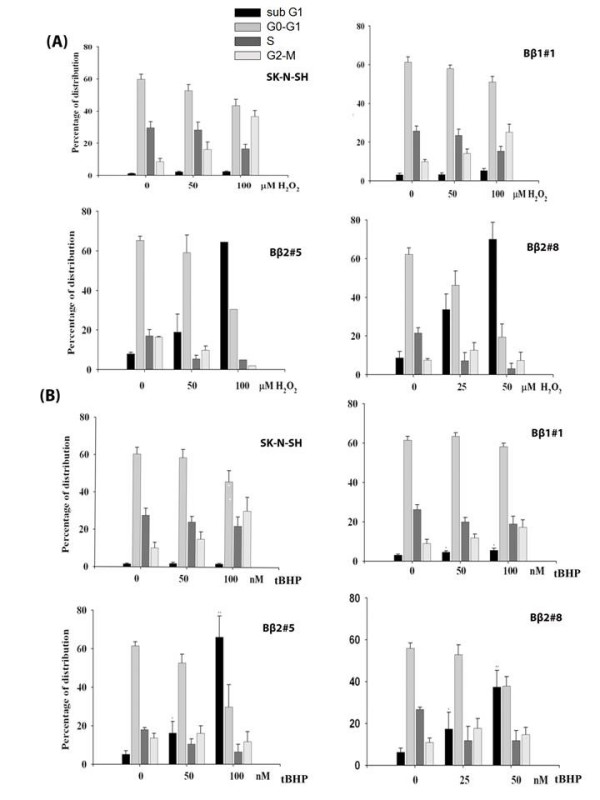
**Both H_2_O_2 _and tBHP increased sub-G_1 _cell populations in different Bβ2 clones as indicated by cell cycle histograms**. Exponential growing cells were cultured with different concentrations of H_2_O_2 _or tBHP for 48 h. The trypsinized cells were analyzed by flow cytometry following staining with propidium iodide. The percentage distribution of cell cycle phases for H_2_O_2 _(A, *top*) or tBHP (B, *bottom*) was determined by Cell Quest software. The results represented averages of three independent experiments and the results were average values in three individual experiments as mean ± standard errors of three independent experiments.

An increase of sub-G_1 _populations was also detected in Bβ2 clones by H_2_O_2 _(Fig 5A) and tBHP (Fig 5B). The results indicated that the decrease of viable cells can be attributed to apoptotic cell death that was not observed in cells with ectopic Bβ1. Thus, it is evident that the apoptotic cell death can be attributed to exogenous ROS in SK-N-SH cells with ectopic Bβ2.

### ROS-induced apoptosis in B*β*2 clones can be rescued by autophagy inhibitor, 3-MA and ATG7 siRNA

**Figure 6:**
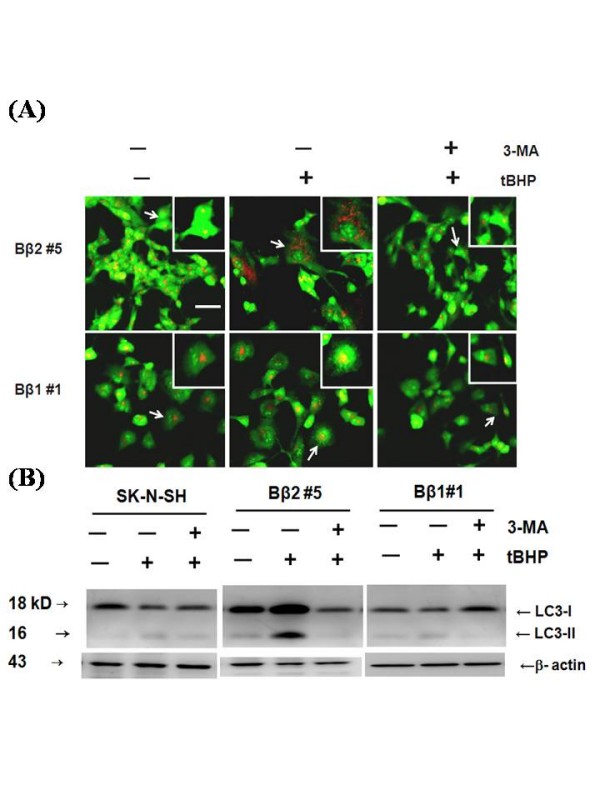
**The inhibitor 3-MA suppressed autophagy development in cells with ectopic Bβ2**. **(A) Autophagosome formation by acridine orange staining**. Cells plated on coverslips in 6-well plates were either pretreated with (+) or without (-) autophagy inhibitor 3-MA before being given 50 nM tBHP for 48 h. The cells were then fixed and stained with acridine orange (*green*) and analyzed by fluorescence microscopy. The formation of autophagosome as shown in puncta fluorescence (*red*) can be suppressed by 3-MA. The presence of autophagy was found mainly in 100% of the cells after treatment, in which more than 100 cells were observed under each condition. *Scales*, 50 μm. Cells as pointed out by arrows were amplified as shown in the inset (2×). (B) **Western blot probed with LC3 antibody **The development of autophagy by tBHP in the presence (+) or absence (-) of 3-MA was analyzed by western blot analysis by incubating the blot with LC3 antibody. Levels of the 18-kDa LC3-I is visualized in both Bβ2 and Bβ1 clones. Elevated LC3-II, the 16-kDa form of LC3 specific for membranes of autophagosomes by tBHP in Bβ2 clones was suppressed by 3-MA.

Autophagic stress can be morphologically recognized by accumulations of autophagic vacuoles in cells [22]. The autophagy marker protein LC3, a mammalian homolog of the yeast Atg8 protein, is covalently modified and redistributes to acidic vesicular organelles (AVO' s) with covalent lipidation as seen by mobility shift from LC3-I to LC3-II by SDS-PAGE. As shown by the acridine-orange staining (Fig 6A) and western blotting with LC-3 antibody (Fig 6B), induction of ROS accelerated cellular changes that are consistent with enhanced autophagy as indicated by the induced formation of AVO' s [25] and the increased lipidation of LC3 (LC3-II) in addition to the basal level. The effect was reverted by the autophagy inhibitor, 3-MA [26].

**Figure 7:**
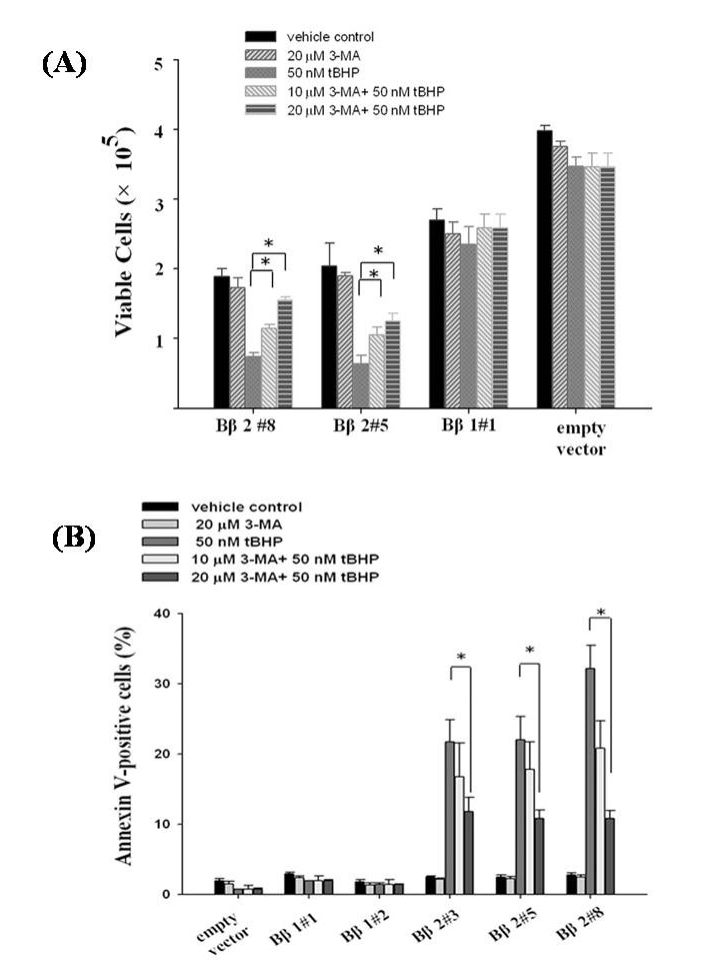
**The autophagy inhibitor rescued tBHP-induced cell death in Bβ2 clones**. (A) **Cell growth determination **A total of 1 × 10^5 ^exponentially growing cells were cultured with either 10 or 20 μM of 3-MA before being treated with 50 nM of tBHP for 48 h. The cells were then trypsinized and counted for viable cells by trypan-blue staining. The results represented averages of three independent experiments and error bars standard errors. (B) **tBHP-induced apoptotic cell death can be blocked by 3-MA **Annexin V binding in Bβ2 clones was determined by flow cytometry 48 h after treatment with 50 nM of tBHP in the presence and absence of 10 or 20 μM of 3-MA, respectively, using SK-N-SH cells as control. The results represented averages of three independent experiments and error bars standard errors. Data are represented as the mean ± standard errors of three independent experiments, each performed in duplicate. *P < 0.05, significance of difference as compared with the control group.

With no distinct affect on cell growth *per se*, 3-MA rescued Bβ2 clones from peroxide-induced cell death (Fig 7A) by suppressing formation of apoptotic cells (Fig 7B) in dose dependent manner. Thus, the accelerated autophagy by peroxide injury antagonized cell survival with supplemented mitochondrial PPP2R2B. The ROS-induced growth inhibition became less evident as cells were pretreated with 3-MA. The addition of 3-MA that rescued apoptotic death in Bβ2 clones reflects the role of autophagy that not only protects cells but initiated apoptotic cell death under oxidative stress.

**Figure 8:**
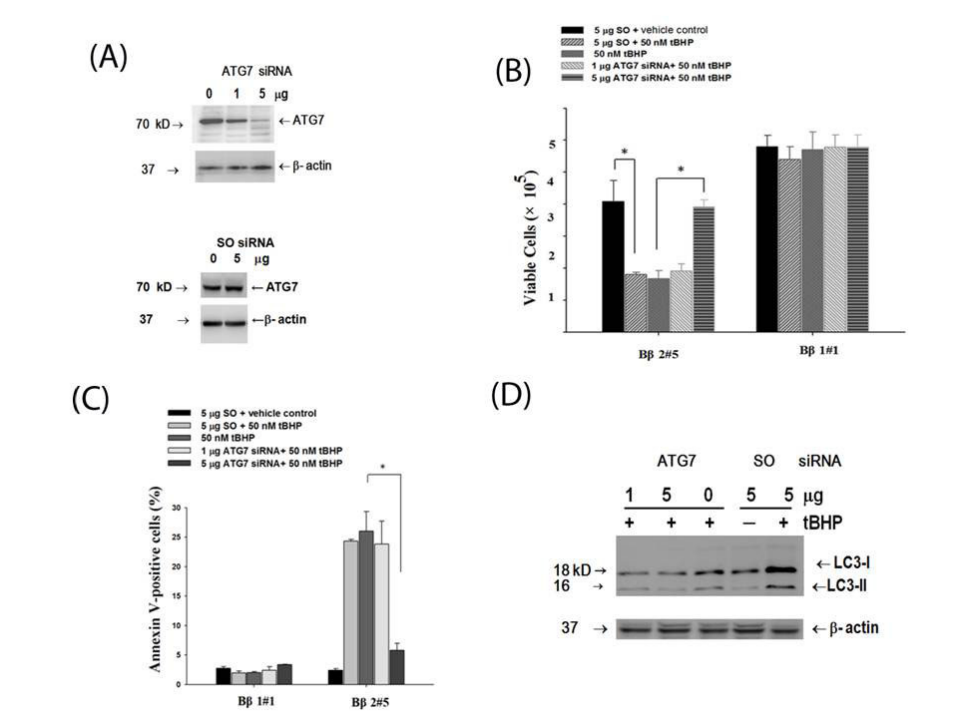
**Transfer of ATG7 siRNA blocked tBHP-induced cell death in Bβ2#5 clone**. A total of 1 × 10^6 ^exponentially growing cells of both Bβ2#5 and Bβ1#1 clones were transfected with either scrambled siRNA (SO) control or ATG7 siRNA, treated with tBHP and analyzed. **(A) Knocked-down ATG5 expression by the increased ATG7 siRNA **Cell lysates of Bβ2#5 clones were probed with ATG7 antibody and reprobed with β-actin antibody to confirm equal loading of the proteins. The blots were then detected with ECL system. **(B) Cell growth **Both Bβ2#5 and Bβ1#1 clones were treated with tBHP for 48 h following transfection with either SO control or ATG7 siRNA. The viable cells were determined by trypan-blue staining. **(C) Determination of apoptotic cells **Distribution of annexin V binding in both clones was determined by flow cytometry after 48 h treatment with 50 nM of tBHP. The results represented averages of three independent experiments and error bars standard errors. Data are represented as the mean ± standard errors of three independent experiments, each performed in duplicate. *P < 0.05, significance of difference as compared with the control group. **(D) Western blot analysis with LC3 antibody **Development of autophagy by tBHP was analyzed by western blot analysis by incubating the blot with LC3 antibody with β-actin antibody as loading control. The elevated LC3-II as visualized the 16-kDa form of LC3 specific for membranes of the formed autophagosomes by tBHP in Bβ2 #5 clones was suppressed by the increased concentrations of ATG7 siRNA.

More work was extended using siRNA-dependent knock-down expression of the essential autophagy gene *ATG7 *[27]. Transfection with ATG7-specific siRNA in Bβ2 clones down-regulated ATG7 expression (Fig 8A), suppressed cell growth inhibition (Fig 8B) and interfered development of apoptosis (Fig 8C) by tBHP, while cells with transfer of the scrambled (SO) siRNA remained unaffected. Moreover, knock-down of ATG7 expression repressed tBHP-induced accumulation of the LC3-II isoform (Fig 8D), that further corroborated autophagy with the development of apoptosis.

## Discussion and Conclusions

PP2A deregulation has been vastly implicated in the onset of neurodegenerative diseases [28]. The mechanisms of PP2A activity responsible for the disrupted neuron function remain unclear and are far from being understood [29]. Intervention of normal brain or cerebellar PP2A function is crucial in the developing neurodegenerative pathology [30]. In an effort to learn how the increased expression of PPP2R2B isoforms affects cell growth, genes encoding proteins encoding different targeting sequences were transferred into human SK-N-SH cells. The clones with ectopically expressed PPP2R2B can be propagated as stable cell lines. Our work showed that discrete antisera cross-reactivity, in which mitochondria can be stained by Bβ1 and cytoplasm by Bβ2, respectively. To understand the function of the ectopic gene in the stable clones, it will be important to further analyze the fractions at subcellular levels.

The stable clones forming aggregation in culture exhibited autophagy characteristics that are distinct from the parental cells. Autophagy is a lysosome-dependent degradation pathway regulated by extracellular nutrients or trophic factors [31]. Cells exhibiting basal rate of autophagy maintain homeostasis and survival of neurons under stress that protects them from pathogenesis [32]. Despite their relatively slow growth rate compared to the parental cells (Fig 7A), clones with ectopic brain-specific subunit PPP2R2B were able to sustain minor increases of autophagosome formation that maintains cell viable. The formation of autophagosome in both Bβ1 and Bβ2 clones (Fig 6) demonstrated how cells survive when encountered with stress conditions. While heterotrimeric PP2A holoenzyme containing all three regulatory subunit families can affect events ranging from the initiation of DNA replication to vertebrate axis formation to apoptosis [8], the up-regulated mitochondrial PPP2R2B putatively serves to deregulate protein kinases cascades, thereby making cells autophagic as seen in some neuron disorders [21,33].

Numerous studies suggested a close relationship between dysfunctional mitochondria and autophagy. Mitochondrial perturbation is one of the sources to induce autophagic cell death [23]. The disturbance of autophagy-lysosome balance has been recognized as a major cause in numerous neurodegenerative disorders [34]. While low levels of autophagy maintains cell viabilities in both stable Bβ1 and Bβ2 clones, only the latter was sensitive to peroxide injury by initiating apoptosis (Fig. 5) with concomitant increase of AVO' s (Fig. 6). Inhibition of autophagy by the chemical inhibitor, 3-MA, (Fig. 7) and RNA interference-mediated knockdown of ATG7 (Fig. 8) reverted the process. Thus, oxidative stress-induced cell death in Bβ2 cells as shown in annexin V staining and growth assays demonstrated that the progression of autophagy lead Bβ2 cells to apoptosis.

Although autophagy is capable of keeping cells from being damaged, the work provided evidence that, in addition to being protective, the increased autophagy as a result of peroxide injury can be pathogenic resulting from apoptosis. Our work showed that the attenuated autophagy prolonged cell survival by reducing formation of the accumulated autophagosome following peroxide injury. Given the fact that oxidative stress is an origin of disturbing cell homeostasis, it is conceivable that the reduced autophagic source may be an alternative approach to ameliorate harmful effects to neuronal system [22]. Being necessary at basal level for maintaining normal axonal and synaptic structures, particularly in Purkinje neurons, excessive autophagy can be neurotoxic and inhibition by chemical regulators prevents the onset of neurodegeneration [27]. Further investigation on how excessive autophagy affects cell signaling and growth under oxidative stress will provide more targets to understand the basis of the diseases.

More evidence indicated that PP2A activity can be regulated by extracellular signals and cell cycle [35]. How altered cell signaling in organelles, particularly those in mitochondria that impinge viable neuron cells as a result of increased expression of the regulatory subunit PPP2R2B, provides an interesting topic for developing more effective preventive measures in development of neuron disorders.

## Methods

### Antisera production

The peptides derived from the N terminus of mitochondria-specific Bβ2 (CFSRYLPYIFRPPNT) and cytoplasm-specific Bβ1(MKCFSRYLPYIFRPPNTILSSSCH) were coupled to keyhole limpet hemocyanin, respectively, via the sulfhydryl group of the N-terminal cysteine; the polyclonal antibody sera were generated in rabbits and purified by standard affinity techniques.

### Cytomegalovirus promoter-driven expression constructs containing Bβ1 or Bβ2 cDNA and establishment of stable clones from SK-N-SH cells and cell culturing

The Bβ1 cDNA was restriction-digested by *Xba *I from the Bβ1 phagemid (provided by BA Hemmings, Katholieke Universiteit te Leuven, Belgium) containing N-terminus cytoplasm-specific sequence of PPP2R2B and ligated into *Xba*I site of pcDNA3. The Bβ2 cDNA containing mitochondria-specific sequence of PPP2R2B was restriction-digested from Bβ2 cDNA library clone by *Xho*I and *Spe*I from the plasmid (provided by S Strack, University of Iowa, Iowa City, Iowa) and ligated into *Xho*I and *Spe*I sites of pcDNA3. Each of the recombinant plasmids was confirmed of their DNA sequences by DNA sequencing from both ends.

Human neuroblastoma cell line SK-N-SH was acquired from American Type Tissue Collection (Rockville, MD) and grown in DMEM (Invitrogen, Grands Islands, NY). Medium was changed three times a week. Cells were observed with a phase-contrast microscope (Nikon Diaphot-300). SK-N-SH cells were cultured every 5 or 7 days using standard trypsinization procedures to maintain the cell line. All cultured cells were supplemented with L-glutamine, sodium pyruvate, and supplemented with 10% heat-inactivated FCS in the humidified atmosphere of 5% CO_2_ at 37°C. All cell lines were examined and found to be free of mycoplasma contamination using a MycoTect kit (Invitrogen, Grands Islands, NY). Both hydrogen peroxide and t-butyl hydroperoxide were from Sigma (St. Louis, MO).

A total of 5 × 10^5 ^exponentially growing SK-N-SH cells were cultured in 60-cm^2 ^flasks and transfected with one microgram of either recombinant B*β*1, B*β*2 construct or the empty vector by Lipofectamine (Invitrogen) in serum-free media following the manufacturer's protocols. After three weeks of culturing and selection with 200 μg/ml of G418 (Invitrogen), the antibiotic-resistant colonies were harvested, and, after limited dilution, the subclones carrying overexpressed Bβ1 or Bβ2 isolated. The established clones can be continuously propagated as immortalized cell lines in 10% FCS-supplemented DMEM.

### Determination for cell growth and apoptosis

Cell growth was measured by trypan blue exclusion assay. Briefly, cells were plated in 12-well culture plates (1 × 10^5 ^cells/well). After incubation for the time specified, the cells were treated with different concentrations of peroxides for the times as specified. The trypsinized cells suspended in PBS were mixed with trypan blue solution in a 1:1 ratio and the cell numbers counted by a hemocytometer.

The annexin V-positive cells were determined using Annexin V-FITC Apoptosis Detection Kit (Roche Diagnostics, Indianapolis, IN) according to the supplier's instructions. Each cell line was tested at least three times and apoptotic cells determined quantitatively by flow cytometry.

### Flow cytometry of cell cycle analysis by propidium iodide staining

To determine phase distribution of DNA content, propidium iodide (PI) staining was performed. Briefly, 3 × 10^5^ cells collected were washed once and fixed in 70% ethanol overnight. After centrifugation at 700 rpm for 5 min at 4°C, cell pellet was stained with 5 μg/ml PI (Sigma, St. Louis, MO) plus 0.5 mg/ml RNaseA in PBS buffer for 15 min at room temperature in the dark. The analysis was performed with FACScan flow cytometer (Becton-Dickinson, Mansfield, MA). Cell cycle distributions were analyzed by Cell-Quest and Modfit software (Becton-Dickinson, Mansfield, MA). The statistics of cell distributions were calculated from three individual experiments.

### PP2A activity assay determination

The phosphatase activity was conducted by modifying the published work [36]. Briefly, cell lysates were immune-precipitated with antiserum against β2, β1 or pre-serum depending on the type of clones used. Following precipitation with protein (A/G)-agarose and resuspension in the assay buffer, the collected supernatants were reacted with p-nitrobenzyl phosphate substrate. The optical readings of the assay as determined by spectrophotometer for each cells were subtracted from those by precipitation with pre-serum. Data were converted into percentage by comparing with those of the control parental cells, SK-N-SH with the empty vector.

### Confocal imaging

Cells were seeded on poly-D-lysine-coated, chambered glasses (20-mm^2^chamber, Nalge Nunc), fixed with 4% paraformaldehyde for 20 min. The cells were incubated with the primary Bβ1 or Bβ2 antiserum followed by TRITC-conjugated secondary anti-rabbit antibody. A concentration 10 nM mitotracker green (Molecular Probes) was added to counterstain mitochondria. Pictures of fixed cells were taken on a Zeiss LSM 510 Meta laser scanning confocal microscope at the Optical Molecular Imaging Microscopy Core Facility, National Taiwan University.

### Transfection of the GFP-LC3 construct and examination of puncta fluorescence

The stables clones Bβ2 and Bβ1 derived from SK-N-SH cells were grown on sterile histologic slides, and, after 24 h, transfected with GFP-LC3 construct (gifted by Dr. Wei-Pang Huang, Department of Life Science, National Taiwan University) using a mixture of Lipofectamine (Invitrogen) and plasmid in Opti-MEM medium (Invitrogen) at a ratio of 5 μL Lipofectamine per milliliter of medium per 1 μL plasmid. After 6 h of incubation, cells were placed in regular complete medium and cultured for 1 day. The slides were washed with PBS, and cells fixed in cold methanol. Cells were then washed in PBS twice, and coverslips mounted with glycerol/PBS (3:1) solution. Slides were examined under a fluorescent microscope (Leica). Autophagy was quantified based on the mean numbers of puncta displaying intense staining for three fields (containing at least 50 cells per field) for each experimental condition. The amount of GFP-LC3 dots, each dot signal was detected by eye and the area was measured using the Photoshop 7.0 software (Adobe).

### Detection of AVO' s by acridine orange staining

Cells were cultured with or without peroxides for 48 hours, washed with PBS, stained with medium containing 0.5 μg/ml of acridine orange for 15 minutes. The media was removed, the cells were washed and fluorescent micrographs taken using an inverted fluorescent microscope.

### Western blot analysis

Cells cultured in 0.5% serum-supplemented media were washed with PBS and scraped in lysis buffer containing 1% triton X-100, 150 mM NaCl, 5 mM EDTA, 1% aprotonin, 5 mM PMSF and 10 μg/ml leupeptin in 20 mM sodium phosphate. Protein concentration was determined by the BCA assay (Pierce Biotechnology, Rockford, IL) and 20 μg of total protein was performed for Western blots analysis. Protein samples were electrophoresed on SDS-polyacrylamide gels, transferred to nitrocellulose filters). The blots wee incubated in fresh blocking solution and probed for 1 h with 1:3,000 dilution of Bβ1 and Bβ2 antisera, respectively. Blots were washed twice in PBS-T and then incubated with a 1:4,000 dilution of peroxidase-conjugated secondary antibody (Kirkegaard and Perry Laboratories, Inc., Gaithersburg, MD) in PBS-T for 1 h at 22°C. Blots were then again washed twice for 10 min in PBS-T and then detected by ECL illumination system (Amersham).

### RNA interference

For siRNA transfection, 1 × 10^6 ^cells were transfected by nucleofection (Applied Amaxa Biosystems) and cell suspensions mixed with 1 or 5 μg of siRNA for Bβ2 cells. The cells were diluted in 2 ml DMEM (supplemented with 10% FBS, 100 U/ml penicillin, 0.1 mg/ml streptomycin, and 2 mM L-glutamine) at 37°C. The siRNA corresponding to the human cDNA sequence for ATG7 (5'-CAGTGGATCTAAATCTCAAACTGAT-3') was acquired from Sigma-Aldrich. The control siRNAs (SO) were acquired from Ambion. The ATG7 signal was verified by Western blotting with the anti-ATG7 rabbit polyclonal antibody (Cell Signalling Technology).

### Statistics

Unless otherwise indicated, results were expressed as compiled means ± S.E. from at least three independent experiments. Values of p < 0.05 were considered significant.

## Authors' contributions

WTC and ZXG participated the biochemical and biology experiments, CAL and MYL conducted isolation of the clones and KF and LCT conceived of the study and research design. KF wrote the paper. All authors read and approved the final manuscript.
